# A Ratiometric pH Sensor for Gram-Positive and Gram-Negative Bacteria

**DOI:** 10.1021/jacs.5c22321

**Published:** 2026-01-20

**Authors:** Dorothea Kossmann, Aya Iizuka, Nina Khanna, Pablo Rivera-Fuentes

**Affiliations:** † Department of Chemistry, 27217 University of Zurich, Zurich 8057, Switzerland; ‡ Department of Biomedicine, 89390 University of Basel, Basel 4031, Switzerland; § Swiss Institute of Bioinformatics, Basel 4051, Switzerland; ∥ Division of Infection Diseases, University Hospital Basel, Basel 4031, Switzerland

## Abstract

Fluctuating environments can lead to phenotypic heterogeneity within a monoclonal bacterial population, especially in response to antibiotics or the human immune system. Methods are required to analyze the physiology of single cells to understand how individual cells interact with their environment and adapt to pH stress. We report a ratiometric, fluorescent probe to sense cytoplasmic pH in bacteria. Our probes are based on hemicyanine dyes and are taken up into both Gram-positive and Gram-negative bacteria. The probes are selective under a broad range of biologically relevant conditions. The response to pH changes is reversible and rapid, allowing for the real-time tracking of pH fluctuations. The sensing of these probes was tuned to allow for monitoring fluctuations around neutrality and biologically relevant acidifications. These probes were validated for cytoplasmic pH sensing in *Escherichia coli*, *Staphylococcus epidermidis*, and a clinically isolated methicillin-resistant *Staphylococcus aureus* (MRSA) strain. Furthermore, the probes enabled the identification of pH-sensitive phenotypes and monitored phagocytosis of virulent clinical strains in immune cells. Our probes are a promising tool for detecting phenotypic heterogeneity within bacterial populations and may help unravel the physiological state of resistant or persistent strains of clinical relevance.

## Graphical abstract

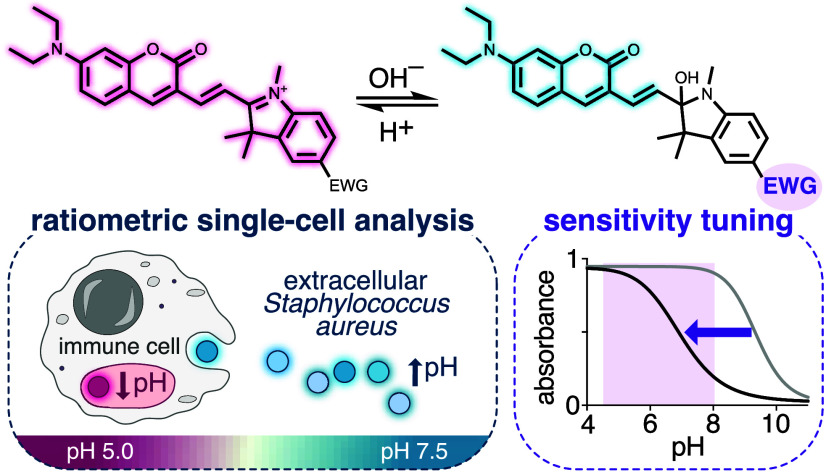

## Introduction

The rise of bacterial antimicrobial resistance is a significant public health threat of the 21st century.^1^ However, it is becoming more evident that even genetically susceptible bacteria may survive antibiotic exposure, and full sterilization is almost never achieved.^2–4^ This antibiotic tolerance differs from resistance in that it does not involve genetic changes, but instead relies on physiological adaptations, such as altered metabolism,^5,6^ growth phase,^7^ and enhanced stress responses.^8^ This phenotypic heterogeneity is modulated by environmental factors, such as heat, acid, antibiotics, and hyperosmotic stress, in addition to stochasticity within biological processes.^9–11^ During infections, the complexity of the host environment induces diverse phenotypic states within genetically identical bacterial populations,^12^ making it difficult to predict individual cell behavior.^13^ Unlike resistance, persistence is difficult to identify due to the absence of reliable markers and the transient nature of underlying physiological states.^4,14,15^ Many phenotype identification methods are time-intensive, such as diffusion assays,^16^ colony growth heterogeneity, and bacterial colonies’ lag times, and do not allow for studying phenotypic changes in real-time.^17,18^ In contrast, fluorescent tools enable rapid, noninvasive single-cell studies, revealing phenotypic heterogeneity in complex biological systems.^19^

Bacterial physiology can be studied on several levels, including gene expression, translation, and metabolism,^20^ but one particularly important physiological parameter is intracellular pH.^21^ It affects protein function, bioenergetics, gene expression, stress responses, virulence, and phenotypic switches.^21–25^ Neutralophilic bacteria like *Escherichia coli* (*E. coli*) tightly regulate their cytoplasmic pH between 7.4 and 7.8,^26–28^ even when exposed to harsh external pH values.^27,28^ The regulatory sensitivity to pH becomes particularly relevant during host–pathogen interactions, as bacteria are often internalized by macrophages into acidic phagosomes.^29^ Acidification aims to degrade bacteria but can unintentionally enhance persistence.^24,30^ Persistence is not solely induced by exposure to low pH. Instead, it has been observed that cells exhibiting a persister phenotype have a more acidic intracellular pH compared to clonal cells that succumb to stress conditions.^31^ This observation suggests that intracellular pH could serve as a potential marker for identifying tolerant cells.

Fluorescent protein sensors, such as the green fluorescent protein (GFP) derivative pHluorin, are widely used to measure bacterial cytoplasmic pH due to their selectivity, brightness, and ability to target specific cellular locations.^31–34^ However, their performance can be affected by buffer composition, bacterial viability, expression levels,^35^ and oxygen availability, which is often essential for chromophore maturation.^36^ Despite extensive refinements, further optimization is often needed for use across diverse bacterial strains.^37–39^ Small-molecule fluorophores offer an alternative, allowing the imaging of nongenetically modified microorganisms, e.g., those isolated from clinical samples.^40^ However, most pH sensors are optimized for mammalian cells, and their uncharged, lipophilic character is poorly suited for bacterial applications since bacteria preferentially take up positively charged, polar, or zwitterionic molecules.^41–43^ Some xanthene-based dyes, such as the cell-permeable acetoxymethyl (AM) esters of fluorescein derivative BCECF (p*K*_a_ = 7.0, pH-sensing range: 6.5–7.5) or SNARF-1 (p*K*_a_ = 7.5, pH-sensing range: 7.0–8.0), have been applied in bacterial imaging, including studies on host–pathogen interactions.^44,45^ Their pH sensing range, however, is narrow and centered near neutrality. More acid-sensitive derivatives like OregonGreen (p*K*_a_ = 4.7, pH-sensing range: 4.0–6.0)^46^ or SNARF-4 (p*K*_a_ = 6.4, pH-sensing range: 6.0–7.5)^47^ are sensitive to acidifications, but display a limited pH range, and might require the use of two pH probes to cover a broader dynamic range.^48,49^ Especially SNARF-4F exhibits reduced bacterial uptake, and is usually used for extracellular pH sensing.^50,51^

To address these limitations, we aimed to develop a bacterial pH sensor capable of sensing pH levels within the physiological range of bacteria, including biologically relevant acidification. Our probe design centered on a coumarin-based dye conjugated to an indoleninium electron acceptor. While a structurally related coumarin-hemicyanine scaffold (CouCy) has previously been reported as a mitochondrial pH sensor, its strong hydrophobic interactions with membranes likely prevent bacterial uptake.^52,53^ Similarly, an aggregation-induced ratiometric pH sensor with a large dynamic range has been reported,^54^ but its high molecular weight and hydrophobic character limit its application in bacterial imaging. Even though a benzooxazine hemicyanine was reported for bacterial imaging, its pH sensing ability is limited to highly acidic (pH 2–4) environments.^55^ Therefore, while a broad array of pH sensors exists for mammalian cell imaging, this repertoire still lacks a ratiometric sensor specifically designed to meet the unique demands of bacterial imaging.

We aimed to fine-tune the pH sensitivity of CouCy scaffolds to achieve a suitable pH-sensing range for visualizing bacterial heterogeneity. Our redesigned probes exhibit properties that favor accumulation in *E. coli* according to the eNTRy guidelines^52^ and allow for live-cell pH sensing experiments. Furthermore, the dynamic range was tuned to cover pH changes around neutrality as well as biologically relevant acidification. The applicability of our probes was validated in both Gram-positive and Gram-negative bacteria and applied to study pH-sensitive *E. coli* knockout strains and host–pathogen interactions of resistant and persistent patient-derived strains.

## Results and Discussion

### CouCy Probes are Selective, Sensitive, and Fast pH Sensors

The CouCy probes **1a**–**c** were synthesized in a condensation reaction of Fischer’s bases **2a**–**c** and coumarin aldehyde **3** (Figure 1A). We titrated the CouCy probes to assess their pH sensitivity (Figures 1B and S1) and calculated the p*K*_a_ values based on the Henderson–Hasselbalch equation (Table S1). We found that the pH sensitivity can be tuned by the substituent on the indoleninium core. The unsubstituted derivative CouCyH **1a** has a p*K*_a_ value of 9.4, whereas the introduction of electron-withdrawing groups (EWG) like the trifluoromethyl (CF_3_) and nitrile (CN) in CouCyCF_3_ **1b** and CouCyCN **1c** lowered the p*K*_a_ to 7.0 and 6.8, respectively. Importantly, these probes maintained their pH sensitivity in a biologically complex mixture containing 10% fetal bovine serum (FBS; Figure S2 and Supporting Information comment 1). Since CouCyCN **1b** and CouCyCF_3_ **1c** exhibit a p*K*_a_ value close to the internal pH range of neutrophilic bacteria, we focused on the further characterization of these probes.

**Figure 1:**
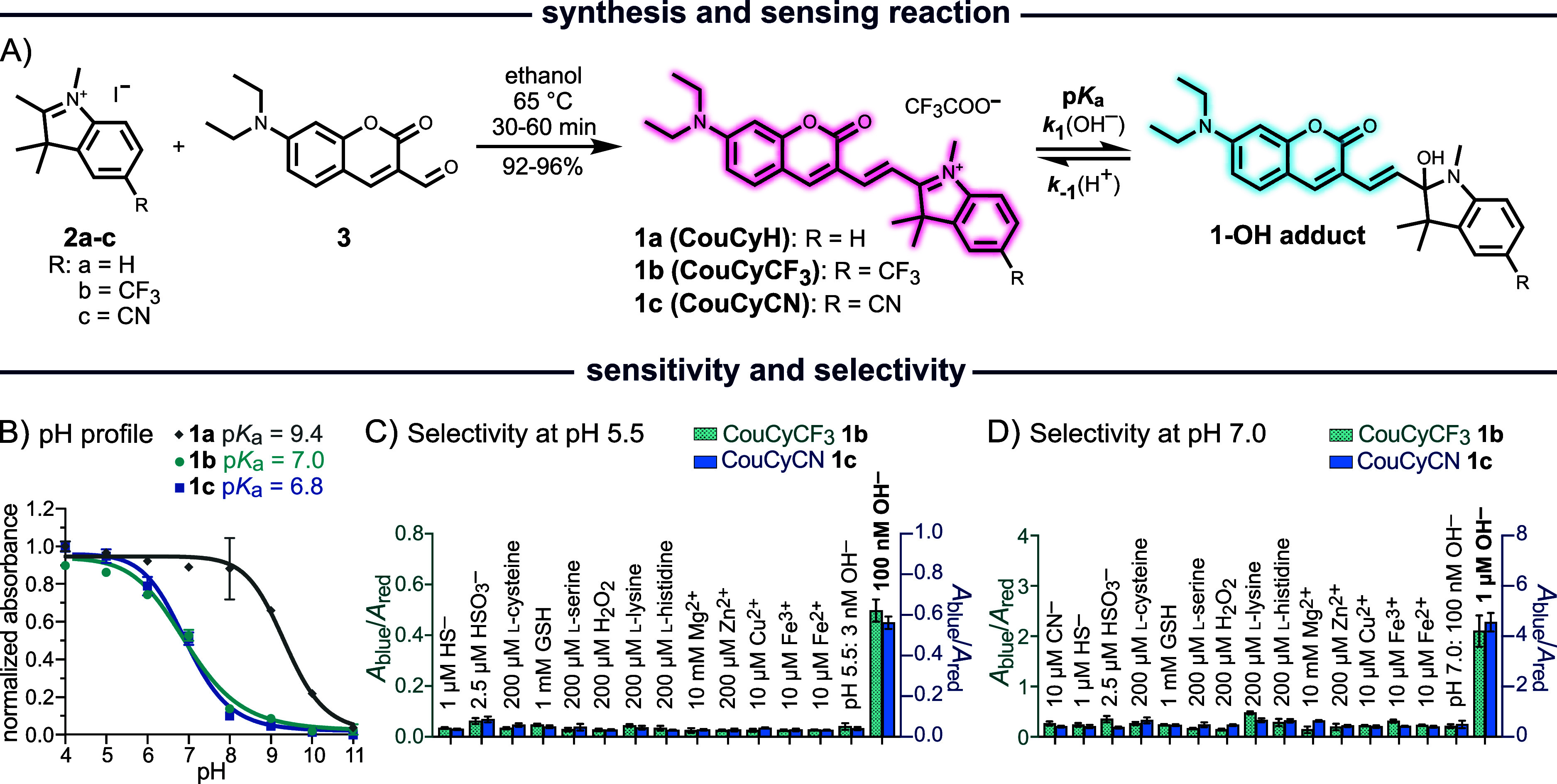
Synthesis and characterization of CouCy’s. (A) Condensation reaction of indoleninium **2a**–**c** and coumarin aldehyde **3** to yield the CouCy derivative **1a, 1b,** and **1c** and equilibrium of the pH sensing reaction. (B) pH profile of CouCyH **1a** (p*K*_a_ = 9.4), CouCyCF_3_ **1b** (p*K*_a_ = 7.0), and CouCyCN **1c** (p*K*_a_ = 6.8). The probe (5 μM) was incubated for 60 min at 37 °C with defined pH values. As pH buffer citric acid and Na_2_HPO_4_ (pH 2–8), NaHCO_3_ and Na_2_CO_3_ (pH 9–11), or NaOH and KCl (pH 12–13) were used. Absorbance was normalized to the absorbance maximum *A*_max_ (**1a**: 570 nm; **1b**: 584 nm; **1c**: 600 nm) and interpolated as sigmoidal curves. The p*K*_a_ values were calculated with the Henderson–Hasselbalch equation $pK_{a}=pH-\left(\log\left(\frac{A_{\max}-A}{A-A_{\min}}\right)\right)$ with *A*_max_ as red absorbance maxima and *A*_min_ as blue absorbance maxima (**1a**: *A*_440_ and *A*_568_; **1b**: *A*_584_ and *A*_415_; **1c**: *A*_600_ and *A*_415_). (C,D) Selectivity assays based on *A*_blue_/*A*_red_ ratio change of CouCyCF_3_ **1b** (green bars; *A*_415_/*A*_585_) and CouCyCN **1c** (blue bars; *A*_415_/*A*_600_) at pH 5.5 (C) and pH 7.0 (D). Absorbance spectra were measured in the presence of 1 μM NaSH, 2.5 μM NaHSO_3_, 1 mM GSH, 200 μM H_2_O_2_, 200 μM l-cysteine, 200 μM l-serine, 200 μM l-lysine, 200 μM l-histidine, 10 μM CN^–^ (only at pH 7.0), 10 mM Mg­(NO_3_)_2_, 200 μM Zn­(NO_3_)_2_, 10 μM Cu­(NO_3_)_2_, 10 μM Fe­(NO_3_)_3_ and 10 μM Fe­(SO_4_)_2_. Probe (10 μM) was dissolved in 10× PBS (pH 5.5 or 7.0) and incubated with the analytes for 30 min at 37 °C and 180 rpm. All data are mean values of triplicates and error bars represent the standard deviation (SD).

Based on structurally similar molecules, we assumed the CouCy scaffold to undergo a nucleophilic attack by hydroxide ions (OH^–^), resulting in a shorter conjugation system as illustrated in Figure 1A.^53^ To investigate the pH sensing mechanism, we measured ^1^H NMR spectra of CouCyCN **1c** in deuterated acetonitrile (CD_3_CN) with NaOD. In the absence of NaOD, a single signal for the two methyl groups (C13) on the indoleninium was observed (Figure S3). After incubation with NaOD, this signal splits into diastereotopic singlets (13′ and 13″), indicating the formation of a stereocenter nearby, with the methyl group (C9) on the indoleninium nitrogen displaying an upfield shift. In addition, the methine groups on C7 and C8 show a *trans*-coupling constant before (^*trans*,3^ *J*_7,8_ = 15.5 Hz) and after NaOD treatment (^*trans*,3^ *J*_7,8_ = 16.0 Hz). Thus, an attack by OH^–^ on the electrophilic Michael acceptor can be excluded. This regioselectivity differs from that of previously reported phosphine nucleophiles, which typically attack CouCy scaffolds at the bridging double bond (C7).^56^ The difference in regioselectivity of the nucleophilic attack might be explained by the Hard–Soft Acid Bases (HSAB) principle. Whereas OH^–^ ions are hard nucleophiles, phosphines (PR_3_) are softer and react with the soft, electrophilic position on C7.^57^ Overall, the NMR data suggest an OH^–^ attack on the electrophilic sp^2^ carbon on the indoleninium, consistent with the literature on a similar scaffold.^53^ Notably, the EWG (R = CN or CF_3_) on the indoleninium influences both the sensitivity to OH^–^ and the regioselectivity of the nucleophilic attack. At high OH^–^ concentrations (pH ≥ 10), the lactone of the coumarin core can be hydrolyzed, resulting in a longer conjugation pathway and red-shifted absorbance (Figure S1).^58^ Such a reactivity was observed with CouCyH **1a**, indicated by a red shift in the absorbance maxima *A*_max_ (pH < 10: *A*_max_ = 570 nm; pH > 10: *A*_max_ = 650 nm; Figure S1). Thus, the OH^–^ regioselectivity depends on the trigonal carbon’s electrophilicity, with more electron-rich CouCy indoleninium prone to undergo a competitive nucleophilic attack on the coumarin lactone. This observation further supports the importance of the electronic tuning introduced with the CN and CF_3_ groups.

To ensure that only one reaction contributes to the pH sensing ability of these probes, we studied the acidification of the diethylamine substituent. This functional group contributes to the donor–acceptor system of the chromophore, enabling intramolecular charge transfer and a strong fluorescence.^59^ Consequently, protonation of the amine group to a nondonating ammonium cation would result in a diminished emission signal. To evaluate this potentially confounding reaction, we performed a low-pH titration of CouCyCF_3_ **1b** (Figure S4) and found that the tertiary amine group is weakly basic, undergoing protonation only under highly acidic conditions (pH < 2; p*K*_a_ = 1.4). This low p*K*_a_ value can be rationalized by the strong electron-withdrawing effect of the conjugated indoleninium acceptor, which reduces the electron density and basicity of the amine. This finding highlights the stability of the CouCy scaffold, which retains its fluorescent properties under strongly acidic conditions.

We investigated the sensing kinetics of CouCyCF_3_ **1b** and CouCyCN **1c** in vitro under pseudo-first-order conditions with an excess of OH^–^ or H^+^, respectively (Figure S5). We found that the OH^–^ attack on CouCyCN **1c**, with an observed pseudo first-order rate constant *k*_1,obs_ = 0.15 s^–1^, is twice as fast as on CouCyCF_3_ **1b**, with a *k*_1,obs_ = 0.09 s^–1^. A similar trend was observed upon changing the pH from 9.0 to 5.0, with CouCyCN **1c** equilibrating faster than CouCyCF_3_ **1b** (Figure S5). The difference in kinetics of CouCyCF_3_ **1b** and CouCyCN **1c** correlates with the strength of the EWG based on the Hammett parameter σ (CF_3_: σ_p_ = 0.54; CN: σ_p_ = 0.66).^60^ Additionally, the reversibility of the pH-sensing reaction was demonstrated by alternating additions of OH^–^ and H^+^ (Figure S6). Overall, these experiments demonstrate that the sensing reaction is reversible and complete within a few minutes, allowing for dynamic monitoring of intracellular pH fluctuations with good time resolution.

To assess the selectivity for OH^–^, we analyzed the ratiometric absorbance change of CouCyCF_3_ **1b** and CouCyCN **1c** in the presence of other nucleophiles at pH 5.5 and 7.0 (Figure 1). We used the ratio *A*_blue_/*A*_red,_ where the blue signal corresponds to the conjugate base CouCy-OH and the red signal to the parent CouCy (conjugate acid). Since the base is formed upon nucleophilic attack, *A*_blue_ in the numerator emphasizes the extent of CouCy-OH formation, and a change in *A*_blue_/*A*_red_ ratio assesses the reactivity with other nucleophiles. The following analytes were used in biologically relevant concentrations or excess: 1 μM HS^–^, 2.5 μM HSO_3_^–^, 1 mM reduced glutathione (GSH), 200 μM H_2_O_2_, 200 μM of the nucleophilic amino acids l-cysteine, l-serine, l-lysine, l-histidine, 10 mM Mg^2+^, 10 μM Cu^2+^, 200 μM Zn^2+^, 10 μM Fe^2+^ and Fe^3+^ and 10 μM CN^–^ (see Suppoting Information for comment on concentration ranges). The selectivity assays revealed that CouCyCF_3_ **1b** and CouCyCN **1c** are selective to OH^–^ ions over other nucleophiles or ions of biological relevance (Figure 1C,D). Based on reported indoleninium-based sensor probes, CN^–^,^61,62^ HS^–^,^63–65^ and GSH^66,67^ are the most competitive nucleophiles to OH^–^. Whereas cyanogenic bacteria like *Pseudomonas aeruginosa* can produce CN^–^ in the μM range, CN^–^ is commonly absent in most bacteria due to its toxicity.^68^ Thus, we assume no interference by CN^–^ ions on our pH sensing in *E. coli*, *Staphylococcus* (*S.*) epidermidis, or *Staphylococcus aureus*.

The reported H_2_S concentration in bacteria is in the low micromolar range (*E. coli*: 3 μM; *S. aureus*: up to 60 μM).^69^ In an OH^–^/HS^–^ competition study, we found that CouCyCF_3_ **1b** is more selective than CouCyCN **1c**. The pH sensing ability of CouCyCF_3_ **1b** remained unaffected in the presence of 10 μM HS^–^ (Figure S7), whereas CouCyCN **1c** undergoes competitive nucleophilic attack of HS^–^ indicated by a change in the *A*_blue_/*A*_red_ ratio. At higher HS^–^ concentrations (≥100 μM), both probes exhibit competitive reactivity, particularly above pH 7.0, where the fraction of deprotonated HS^–^ increases. Given that physiological concentrations of H_2_S are unlikely to surpass 100 μM and our probes are largely intended to be used for sensing acidification, the interference of HS^–^ in intracellular pH sensing is negligible. In a second thiol-pH competition study, we assessed the reactivity of CouCy dyes with GSH. In most Gram-negative bacteria, GSH is present in the mM range, but is absent in Gram-positive species.^69^ We found that below pH 8.0 and at concentrations from 0.01 to 5 mM, GSH had a minimal impact on the ratiometric signals of CouCyCF_3_ **1b** (Figure S7). In contrast, CouCyCN **1c** showed an increased susceptibility to nucleophilic attack by GSH, as previously described for HS^–^. These measurements revealed that CouCyCF_3_ **1b** is more selective for pH sensing and indicate under which conditions the effect of HS^–^ and GSH fluctuations could be safely ignored, which is mostly at acidic pH (pH < 7.0).

Sulfur dioxide (SO_2_), which in water is in equilibrium with the hydrated forms bisulfite and sulfite (HSO_3_^–^/SO_3_^2–^) may be generated in bacteria with a sulfur-based energy metabolism.^70^ Both HSO_3_^–^/SO_3_^2–^ are nucleophilic and reducing agents, and they are expected to react with electrophiles such as CouCy probes. We therefore tested this reactivity in a competition assay as described for HS^–^ and observed that at concentrations above 5 μM, HSO_3_^–^/SO_3_^2–^ can disrupt pH sensing considerably (Figure S7). Such concentrations are unlikely to occur in pathogenic bacteria of human interest that lack or have downregulated sulfate reduction pathways (e.g., *E. coli* and *S. aureus*).^71,72^ However, high concentrations of HSO_3_^–^/SO_3_^2–^ may occur in bacteria with dedicated biosynthetic pathways for HSO_3_^–^/SO_3_^2–^, such as the dissimilatory sulfate reduction^70^ and assimilatory sulfate reduction^73^ pathways. CouCy probes are not suitable for monitoring pH changes in those species.

### CouCy Sensors Reliably Detect pH Changes in Live Gram-Negative and Gram-Positive Bacteria

To evaluate the pH-sensing capability of CouCyCF_3_ **1b** and CouCyCN **1c** in live cells, we performed intracellular pH calibration experiments using *E. coli* and *Staphylococcus epidermidis*. The bioavailability of small-molecule probes in bacteria depends on uptake, distribution, and efflux pathways.^42^ Especially, staining of Gram-negative bacteria is challenging due to the permeability barrier of the outer membrane.^74^ However, the CouCy scaffold has physicochemical properties that favor accumulation in *E. coli*, like a small size (<600 Da), a positive charge, low globularity (≤0.25), and rigidity due to the conjugation system (Table S2).^52^ Furthermore, the dual-wavelength response of CouCy probes enables ratiometric fluorescence measurements, providing a readout that is independent of probe concentration and instrument fluctuations, thereby increasing accuracy and sensitivity.^75^ Such self-calibration is particularly advantageous for bacterial imaging, where the bioavailability of small-molecule probes is modulated by uptake and efflux and may fluctuate depending on the bacteria’s environment and physiological state.^42^

We used *I*_red_/*I*_blue_ for ratiometric analysis, as it provided a broader dynamic range compared to the inverse *I*_blue_/*I*_red_ ratio and exhibits better sensing ability in the desired acidic range with an improved signal-to-noise ratio, as the signal of the conjugate acid (parent CouCy) dominates the ratio (Figure S8).

To evaluate the uptake efficiency, we incubated the Gram-negative *E. coli* K12 with 5 μM, 2 μM, or 1 μM CouCyCF_3_ **1b** and found strong intracellular staining at all concentrations (Figure S9), suggesting efficient intracellular accumulation. The ratiometric signal remained constant across concentrations, demonstrating the self-calibration and concentration independence of our probes (Figure S9). Additionally, we analyzed the time-dependent staining of *E. coli* with 1 μM CouCyCF_3_ **1b** using flow cytometry (Figure S10). Bacterial staining was observed immediately, and the ratiometric readout remained constant for 30 min, with only minor fluctuations. This data suggests that the molecule is stable under physiological conditions and that increased efflux or aggregation is negligible. This observation is further supported by a long-term stability study under simulated physiological conditions using PBS buffer supplemented with a protein content similar to that in the bacterial cytoplasm.^76^ Under such conditions, the *I*_red_/*I*_blue_ ratio of CouCyCF_3_ **1b** fluctuated only slightly, even as signal intensity decreased over 48 h (Figure S11).

Despite weak emission signals in aqueous medium, we observed a good intracellular signal, suggesting some degree of fluorogenicity. We propose that the intracellular fluorogenicity might be related to the environment-dependent photophysical properties of CouCy dyes, which exhibit low quantum yields in aqueous media but increased emission in apolar intracellular environments (Figure S9 and Table S3). Weak emission signals in polar media may result from twisted intramolecular charge-transfer (TICT) states, which involve bond rotation and geometric distortion. TICT processes are modulated by solvent polarity, viscosity, and temperature.^59,77^ To investigate these effects, we characterized the spectral properties of CouCyCF_3_ **1b** and CouCyCN **1c** across a range of organic solvents with varying polarity and viscosities.

CouCys have extinction coefficients ε (**1b**: 72 × 10^3^ M^–1^ cm^–1^; **1c**: 51 × 10^3^ M^–1^ cm^–1^ at pH 7.4) that are comparable to those of other pH-sensors such as SNARF-4F (48 × 10^3^ M^–1^ cm^–1^ at pH 9) or BCECF (90 × 10^3^ M^–1^ cm^–1^ at pH 9).^78^ However, the emission quantum yield ϕ_em_ (**1b:** 0.8%;^56^ **1c**: 1.4%) and resulting brightness ε × ϕ_em_ (**1b**: 574 M^–1^ cm^–1^; **1c**: 715 M^–1^ cm^–1^) of CouCy dyes in aqueous solution are rather low (Table S3).

Upon decreasing solvent polarity, we observed a bathochromic shift, a narrowing of the red emission maxima, and an increase in the emission intensity compared to aqueous medium (Figures 2 and S12). Such solvatochromic properties are well-known for coumarin-based chromophores in which TICT states likely have stronger H-bonding capabilities and a larger dipole moment than the emissive excited state. Therefore, as solvent polarity decreases, TICT states are destabilized and the emissive excited state is stabilized, resulting in enhanced signals with a bathochromic shift.^59,77^ Consequently, the extinction coefficients and quantum yields of CouCy probes depend on solvent polarity (Figure S13 and Table S3) and the resulting brightness ε × ϕ_em_ increases as solvent polarity decreases, as observed for acetonitrile (**1b**: 1115 M^–1^ cm^–1^; **1c**: 1012 M^–1^ cm^–1^) and dichloromethane (**1b**: 14,814 M^–1^ cm^–1^; **1c**: 11,206 M^–1^ cm^–1^).

**Figure 2:**
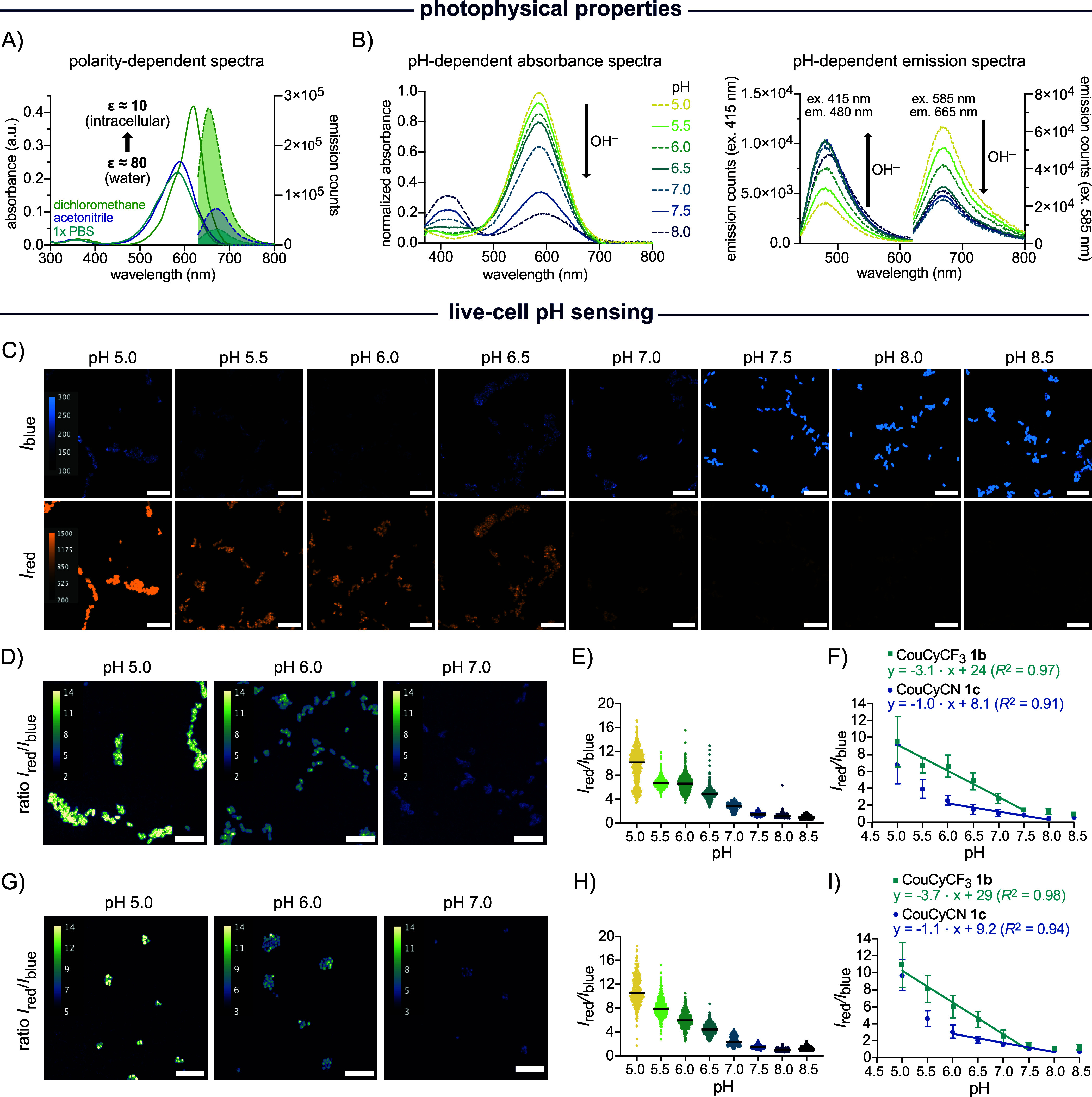
In vitro characterization and microscopy images of live-cell pH calibration experiments. (A) Polarity-dependent absorbance (bold lines; 2.5 μM probe) and emission spectra (dashed lines with filled area; 1.0 μM probe) of CouCyCF_3_ **1b** in 1× PBS pH 7.4 (ε = 80), acetonitrile (ε = 38), and dichloromethane (ε = 9).^88^ (B) CouCyCF_3_ **1b** pH-dependent absorbance spectra (left; 5 μM probe) and emission spectra (right; 1 μM probe) with two *y*-axis (ex. 415 nm and ex. 585 nm). All spectra were measured in quartz cuvettes, were background-corrected, and represent the mean of preparative triplicates. (C) Confocal microscopy images of *E. coli* treated with CouCyCF_3_ **1b** (1 μM) for 20 min at 37 °C, followed by a CCCP (250 μM) treatment in PBS (10×, varying pH) for 60 min at 37 °C. (D) Representative ratiometric images of *E. coli* treated with CouCyCF_3_ **1b** (1 μM) and CCCP (250 μM) at pH 5.0, 6.0, and 7.0. (E) Scatter plot of *I*_red_/*I*_blue_ (*I*_640_/*I*_445_) ratio from *E. coli* stained with CouCyCF_3_ **1b** (1 μM), with *N* = 499, 683, 771, 747, 545, 762, 734, 545 (from left to right), independent single cells or cell clusters from two separate imaging sessions. (F) Linear regression of the *I*_red_/*I*_blue_ (*I*_640_/*I*_445_) mean with SD in *E. coli* K12 stained with CouCyCF_3_ **1b** (1 μM) or CouCyCN **1c** (1 μM). (G) Representative ratiometric images of *S. epidermidis* treated with CouCyCF_3_ **1b** (1 μM) and CCCP (250 μM) at pH 5.0, 6.0, and 7.0. (H) Scatter plot of *I*_red_/*I*_blue_ (*I*_640_/*I*_445_) ratio with *N* = 238, 352, 388, 339, 234, 310, 189, 220, independent single cells or cell clusters from two separate imaging sessions. (I) Linear regression of the *I*_red_/*I*_blue_ (*I*_640_/*I*_445_) mean with SD in *S. epidermidis* stained with CouCyCF_3_ **1b** (1 μM) or CouCyCN **1c** (1 μM). Imaging was performed with the following laser setup: ex. 445 nm, em. 472/30 nm, 1.8 mW, 400 ms; ex. 640 nm, em. 708/75 nm, 3.8 mW, 400 ms. Scale bar, 10 μm.

Additionally, we observed that both probes exhibit an increase in blue emission and a decrease in red emission intensity as the polarity decreases. Interestingly, in very apolar media, which cover the polarity range of bacteria (dielectric constant ε < 10),^79–81^ CouCyCN **1c** exhibits intense blue and almost no red emission intensities, whereas CouCyCF_3_ **1b** shows intense blue and a moderate red emission intensity. This finding suggests that CouCyCF_3_ **1b** exhibits photophysical properties that are advantageous for bacterial imaging. This effect, albeit useful for imaging, could pose a challenge for pH quantification if the brightnesses of the hydroxylated and parent CouCy dyes varied strongly with polarity. Fortunately, we found that this effect is negligible within the polarity range of bacteria, as the ratiometric readout *I*_red_/*I*_blue_ remains constant in highly apolar environments (Figure S14 and Supporting Information comment 2).

Since structurally similar dyes have been reported to change their spectral properties with variations in viscosity,^59,82,83^ we investigated the pH-sensing abilities of the CouCy probes in water/glycerol mixtures (Figure S15). Notably, both CouCyCF_3_ **1b** and CouCyCN **1c** maintained their pH-dependent ratio change across viscosities. Additionally, we observed an overall increase in signal intensity and a larger pH-dependent *I*_red_/*I*_blue_ ratio change at higher viscosities, suggesting an increased sensitivity. This increased pH-sensitivity could be beneficial, provided that intracellular viscosity is higher than water and remains relatively constant across the biologically relevant pH range. Despite the macromolecular crowding and high protein concentration, the cytoplasmic space is only about 2–3 times more viscous than water, as reported for *E. coli* (2.82 ± 0.42 mPa·s).^84^ Therefore, we expect only minor viscosity-dependent contributions to our ratiometric pH-sensing in bacteria. To further evaluate this assumption, we determined whether cytoplasmic viscosity varies across the physiologically relevant pH range, using the established viscosity sensor thioflavin-T (Figure S16).^85,86^ To conduct intracellular pH calibration in live cells, we used carbonyl cyanide *m*-chlorophenyl hydrazone (CCCP; 250 μM), a chemical inhibitor of oxidative phosphorylation and a mediator of H^+^ influx that uncouples the cellular proton gradient.^87^ CCCP allows to equilibrate the extracellular and intracellular pH, bypassing the ability of bacteria to tightly regulate their intracellular pH.^27,28^ Under these conditions, the viscosity-dependent emission signal (λ_ex_ = 445 nm) of thioflavin-T showed no systematic correlation with intracellular pH changes, indicating that cytoplasmic viscosity remains stable across the physiologically relevant pH range (Figure S16).

Having established that neither polarity nor viscosity changes interfere with the signal of CouCy probes during intracellular pH changes, we conducted pH-calibration experiments with our CouCy probes directly in live cells to assess their pH-sensing performance in a biologically relevant sample (Figures 2 and S8). Live-cell fluorescence microscopy of *E. coli* K12 stained with CouCyCF_3_ **1b** (Figure 2C) showed a pH-dependent emission shift: under acidic conditions, fluorescence was mainly observed in the red channel (λ_ex_ = 640 nm), whereas increasing pH led to a shift toward blue emission (λ_ex_ = 445 nm). Ratiometric images at pH 5.0, 6.0, and 7.0 (Figure 2B) demonstrate clear pH-dependent changes at the single-cell level. Quantitative analysis of the *I*_red_/*I*_blue_ ratio revealed a linear response between pH 5.0 and 7.5 (Figure 2C,D), based on two independent biological replicates. Furthermore, we used *S. epidermidis* as a model organism to evaluate the pH sensing of CouCyCF_3_ **1b** in Gram-positive strains. Confocal images showed similar pH-induced emission shifts (Figure S17), and the ratiometric images confirm a linear *I*_red_/*I*_blue_ response from pH 5.0 to 7.5 (Figure 2E–G), consistent with the results in *E. coli*. These results demonstrate that CouCyCF_3_ **1b** can be employed to sense pH in both Gram-negative and Gram-positive bacteria.

CouCyCN **1c** was also evaluated in both *E. coli* K12 and *S. epidermidis*. Ratiometric fluorescence analysis revealed linear pH-sensing between pH 6.0 and 8.0 in both microorganisms (Figures 2 and S18). Notably, the calibration slopes differ significantly, with CouCyCF_3_ **1b** showing steeper slopes (*m* = −3.1 and −3.7) compared to CouCyCN **1c** (*m* = −1.0 or −1.1), indicating higher sensitivity of CouCyCF_3_ **1b** to pH changes. Furthermore, the linear pH-sensing range of CouCyCF_3_ **1b** extended to pH 5.0. This difference in dynamic range was further highlighted in pH sensing experiments using flow cytometry. *E. coli* K12 were incubated with either probe (1 μM) in the presence of CCCP (250 μM) across pH 5.0 to 8.0. The bacterial populations stained with CouCyCF_3_ **1b**, showed a clear pH-dependent separation, whereas samples stained with CouCyCN **1c** showed less distinct separation across the same pH range (Figure S19). The enhanced dynamic range and increased sensitivity of CouCyCF_3_ **1b** suggest its suitability for high-throughput applications such as fluorescence-activated cell sorting (FACS) to visualize single-cell phenotypes in the analysis of multiple cells. The superior performance of CouCyCF_3_ **1b** may be attributed to its photophysical properties, particularly the polarity-dependent emission intensities, which exhibit strong emission signals at both emission maxima within the polarity range of bacterial cells (Figure S14).

To assess the performance of our probes relative to established pH sensors, we conducted experiments using reported ratiometric pH sensors with comparable p*K*_a_ values. We chose the ratiometric biosensor mCherry-pHluorin, which combines the pH-insensitive mCherry with the pH-sensitive GFP derivative pHluorin. We utilized the plasmid pSCM001 (Addgene: 124605),^34^ which encodes the mCherry-pHluorin fusion protein under an arabinose-inducible promoter. Confocal microscopy images (Figure S20) show the expected pH-induced trend, characterized by increasing pHluorin emission (λ_ex_ = 488 nm) while mCherry emission (λ_ex_ = 561 nm) remained constant.^34^ Ratiometric analysis of *I*_green_/*I*_orange_ revealed a linear pH sensing trend between pH 6.5–8.0 as reported in the literature (Figure S20).^31,34^ Notably, CouCyCF_3_ **1b** showed a larger dynamic range (pH 5.0–7.5), better sensing ability at low pH, and higher sensitivity indicated by a larger slope (*m* = 3.1) compared to mCherry-pHluorin (*m* = 2.2).

In addition to the protein sensor, we conducted experiments with the commercial small-molecule probe BCECF-AM with a p*K*_a_ of 6.9 (Figure S21).^89^ This compound has been successfully applied in bacterial imaging, including studies on host–pathogen interactions.^44,45^ We stained *E. coli* K12 with BCECF-AM (1 μM) and found a linear *I*_green_/*I*_blue_ ratio change between pH 6.5 and 8.5 (Figure S21). The fluorescein derivative showed a high intracellular emission but high background signals, likely resulting from spontaneous hydrolysis in aqueous solutions.^90^ Furthermore, BCECF displays a narrower sensing range than CouCyCF_3_ **1b**. BCECF is an excitation ratiometric probe (λ_ex_ = 490 and 440 nm) with a single emission readout at 510 nm, and its pH-sensitive emission change of xanthene-based dyes is based on the protonation of the xanthene core. The dianion, as conjugate base, is the main fluorescent species, whereas the protonated, conjugate acid species exhibits a weaker donor–acceptor system with diminished fluorescence.^78,91^ In contrast, our CouCyCF_3_ **1b** is an emission ratiometric probe with two distinct excitation and emission wavelengths and exhibits strong fluorescence from both its conjugate acid and base, as the molecule functions as two interconverting fluorophores, each dominating at different pH values. This feature enables ratiometric sensing with higher emission signal intensity across a larger range.

In addition, we conducted pH sensing experiments with the commercial small molecule SNARF-4F-AM (p*K*_a_ = 6.4), which exhibits a more acidic pH sensing range (pH 6.0–7.5).^47^ However, the low emission intensity observed indicated insufficient uptake of the probe (Figure S22), which is not surprising, as SNARF-4F is primarily used for measuring extracellular pH in bacterial studies.^50,51^ This finding highlights that efficient bacterial uptake and retention are crucial but remain a limitation of many small-molecule probes. In conclusion, in comparison to commonly used small-molecule pH sensors, our probes exhibit good bacterial accumulation, a broader dynamic range, and increased sensitivity to detect pH changes.

### CouCyCF_3_ Detects Decreased Acid Tolerance in Bacteria Lacking Cyclopropane Fatty Acids

Cyclopropane fatty acids (CFAs) are linked to stress protection and acid tolerance of bacteria.^92–94^ These fatty acids are synthesized from monounsaturated fatty acids catalyzed by the CFA synthase (cfaS) as a postsynthetic modification of the phospholipid bilayer (Figure 3A). Elevated CFA levels have been associated with increased membrane fluidity and enhanced acid tolerance in *E. coli* strains.^92,93,95^ Conversely, bacteria that lack CFA in their membranes exhibit increased proton permeability.^94^ Previous studies have assessed the acid sensitivity of CFA synthase knockout (Δ*cfaS*) strains using microelectrode-based H^+^ flux measurements^94^ or survival studies based on colony formation on agar plates.^92,93^ We investigated whether our pH probe could visualize the different phenotypes of Δ*cfaS* knockout and parental *E. coli* BW25113 under stress conditions. The acid shock was performed by incubating the cells stained with CouCyCF_3_ **1b** (1 μM) in lysogeny broth (LB) at different pH values (7, 5, 4, and 3).^92,93^ The change of cytoplasmic pH was monitored over time by flow cytometry, allowing for gating acidified cells based on the red (*I*_red_ > 3.5 × 10^3^) and blue (*I*_blue_ > 1 × 10^3^) emission channels (Figure S23). Consistent with literature reports,^27,28^ both strains maintained a stable intracellular pH under mildly acidic conditions (pH 5 or 4), as less than 1% of the cells were acidified (Figure S23). However, exposure to pH 3 resulted in acidification of the cytoplasm, consistent with lethal acid shock conditions (Figure 3B).^92,93^ Importantly, the Δ*cfaS* *E. coli* population undergoes a more significant acidification compared to the parental strain, as indicated by the appearance of a more dominant population with an increased *I*_red_/*I*_blue_ emission ratio (Figure 3B,C). These results confirm the increased sensitivity of Δ*cfaS* strains to acid stress and demonstrate that CouCyCF_3_ **1b** enables real-time visualization of pH-sensitive phenotypes at the single-cell level.

**Figure 3:**
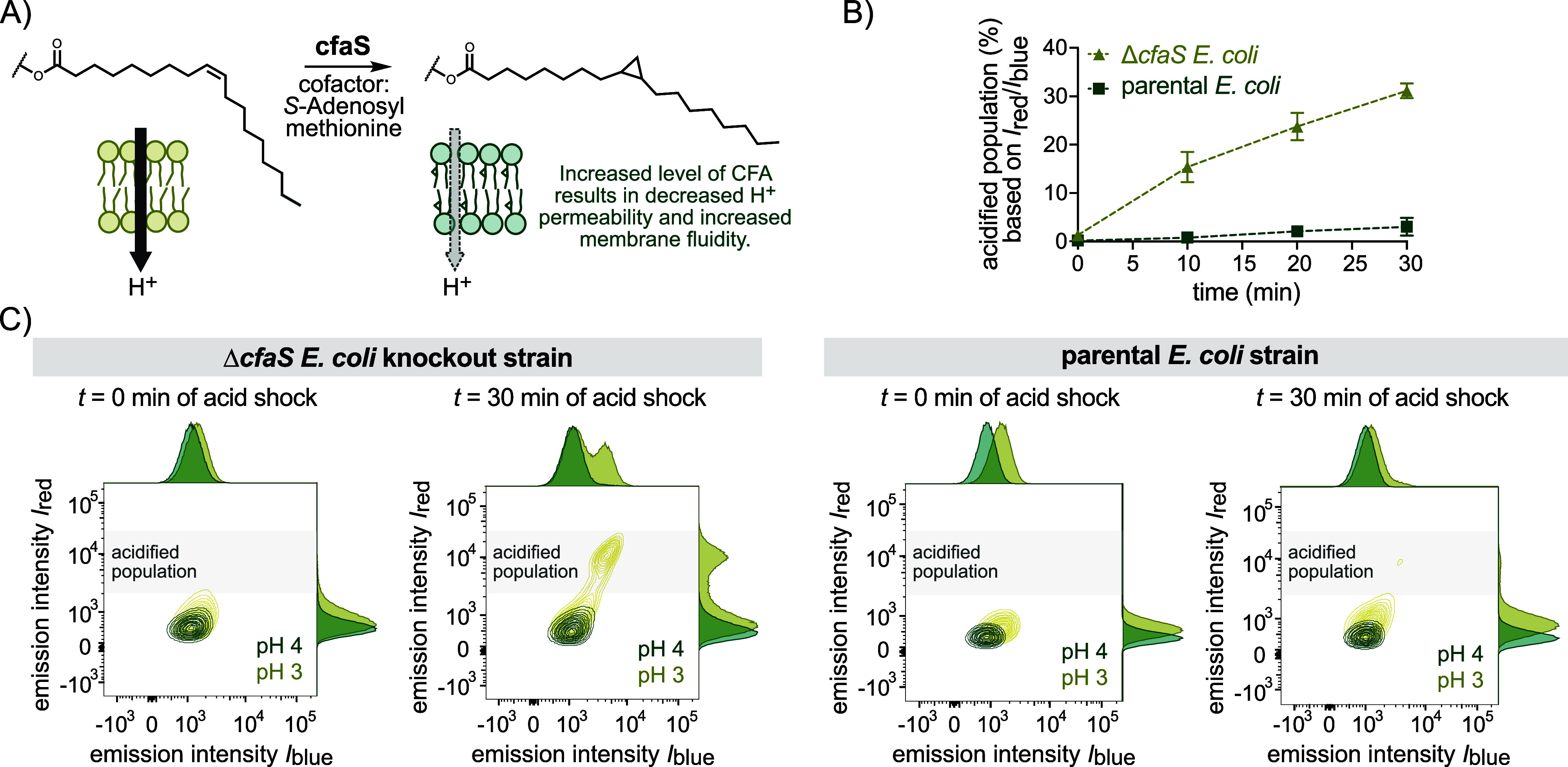
Comparison of pH-sensitive Δ*cfaS* *E. coli* knockout strain with the parental *E. coli* BW25113 (Keio Knockout Collection).^96,97^ (A) Reaction scheme of the cfaS-catalyzed synthesis of CFA using monounsaturated fatty acids and *S*-adenosyl methionine as cofactor. CFA leads to a lipid bilayer with decreased packing density and increased membrane fluidity, resulting in lower proton membrane permeability.^94,98^ (B) Quantitative analysis of Δ*cfaS* (beige) and parental *E. coli* (green) population under acid shock at pH 3 over 30 min stained with CouCyCF_3_ **1b**. Acidified cells were gated based on emission intensity *I*_red_ (*I*_639_ > 3.5 × 10^3^) and *I*_blue_ (*I*_405_ > 1 × 10^3^). Data points are means with SD from two biological replicates. (C) Flow cytometry analysis representing bacterial populations as contour plots and adjunct histograms at time points 0 and 30 min of the acid shock at pH 3 and 4. Each sample was gated for single bacterial cells. Flow cytometry was performed with the following laser setup: ex. 405 nm, em. 515/20 nm (*I*_blue_); ex. 639 nm, em. 670/30 nm (*I*_red_).

### CouCy Probes Can Track Bacterial Acidification in Host–Pathogen Interaction Studies

To answer clinically relevant questions, phenotypes should be studied under in-patient-like conditions, as standard laboratory settings may not accurately replicate virulent phenotypes.^12,20,99^ In this context, we evaluated the applicability of the CouCy probes for host–pathogen interaction studies using the laboratory strain *S. epidermidis* and clinical isolates of *S. aureus*. Specifically, a methicillin-resistant *S. aureus* (MRSA, t619915) strain isolated from a prosthetic joint infection, and a methicillin-sensitive *S. aureus* (MSSA, P70) strain from a deep-seated infection and bacteremia were collected from patients at the University Hospital Basel, Switzerland. The clinical *S. aureus* was efficiently stained with CouCyCF_3_ **1b** and CouCyCN **1c**, allowing for the visualization and quantification of intracellular pH using fluorescence microscopy or flow cytometry (Figure S24). Additionally, we assessed the impact of CouCy dyes on bacterial viability. At the working concentration of 1 μM, no cytotoxic effects were observed, as bacterial growth remained unaffected over 24 h. Higher concentrations (5 μM) induced only a minor reduction in bacterial growth rate (Figure S25), indicating that CouCy dyes are well tolerated under the experimental conditions used.

For infection studies, the laboratory strain *S. epidermidis* or clinical isolates of *S. aureus* were prestained with CouCyCF_3_ **1b** (1 μM) before coculturing with THP-1 monocytes in serum-containing medium (RPMI + 10% FBS). A multiplicity of infection (MOI) of 3 was induced to achieve 3× more bacterial cells than immune cells. Intracellular bacteria were detected 10 min postinfection using confocal microscopy (Figure S26) or imaging-based flow cytometry (ImageStream; Figure S27). The increased *I*_red_/*I*_blue_ emission ratio indicates that intracellular bacteria are experiencing acid stress in phagolysosomes, whereas extracellular bacteria maintain their intracellular pH neutral, with a lower *I*_red_/*I*_blue_ ratio. This experiment demonstrates that CouCyCF_3_ **1b** enables the detection of extracellular and intracellular bacteria and can be used to study phagocytosis in immune cells. A widely used fluorescent dye for monitoring phagocytosis is pHrodo Red,^100^ which exhibits increased emission at low pH and has a broad dynamic range (pH 4–8). Despite its utility, pHrodo Red provides only a single-channel readout, limiting its application to qualitative assessments.^101^ In contrast, the ratiometric properties of CouCyCF_3_ **1b** enable quantitative pH measurements, facilitating comparative studies. Thus, we conducted a quantitative intracellular pH comparison study as part of our host–pathogen interaction experiment.

To mimic in-patient-like conditions accurately, blood cells from freshly heparinized human whole blood were extracted and cocultured with clinical *S. aureus* strains. The obtained blood cell samples represent a biologically complex mixture, including monocytes, neutrophils, and lymphocytes. As previously demonstrated for the THP-1 cell line, we were able to identify extracellular and intracellular bacteria 10 min postinfection (Figures 4A and S28). With flow cytometry, extracellular single cells could be easily gated for size by scattering. However, we observed that sometimes bacteria appear bound to immune cells even though they have not been internalized, resulting in a single event that can hardly be identified through scattering or in the brightfield image of imaging-based flow cytometry. CouCyCF_3_ **1b** allows for the visualization of these extracellular bacterial cells (Figures 4A and S28), providing an additional feature to track preinfection events.

**Figure 4:**
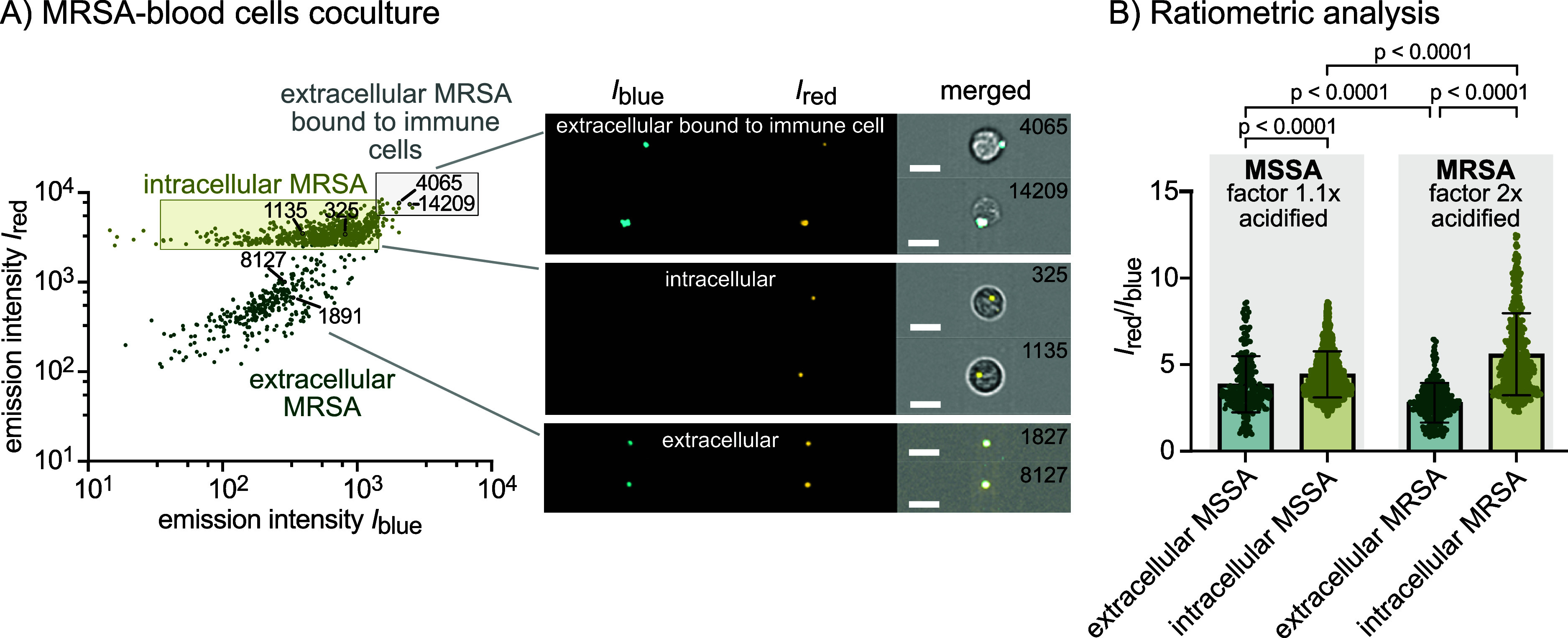
Phagocytosis study with clinical isolates of *S. aureus* and blood cells. (A) Scatter plot and images of extra- and intracellular MRSA cells stained with CouCyCF_3_ **1b** (1 μM) 10 min postinfection. Internalized bacteria (yellow; events 1135 and 325) in blood cells undergo phagocytosis, represented as a population shift to higher red emission intensities compared to the extracellular bacteria population (green; events 1827 and 8127). Preinfection events of extracellular bacteria bound to immune cells appeared with higher blue and red emission intensities (events 4065 and 14209). Merged images display the overlay of the brightfield and emission channels. Flow cytometry was performed with the following laser setup: ex. 405 nm, em. 435–505 nm (*I*_blue_); ex. 642 nm, em. 642–745 nm (*I*_red_). Scale bar, 7 μm. (B) Quantification of internal pH based on the *I*_red_/*I*_blue_ (*I*_642_/*I*_405_) emission intensities. Graph represents single cell intensities (scatter plot) and means (bar plot) with SD (error bar) for extracellular MSSA (mean = 3.9 ± 1.6, *N* = 270), intracellular MSSA (mean = 4.4 ± 1.3, *N* = 2246), extracellular MRSA (mean = 2.8 ± 1.1, *N* = 325), and intracellular MRSA (mean = 5.6 ± 1.1, *N* = 553). Statistical significance was evaluated using the Kruskal–Wallis test (multiple comparisons). Outliers were identified and removed using the ROUT method (*Q* = 1%) with 5%, 6%, 1%, and 12% outliers (from left to right).

Our quantification of the *I*_red_/*I*_blue_ ratio showed that extracellular MSSA cells have a more acidic intracellular pH compared to MRSA (Figure 4B). Interestingly, this trend was reversed upon phagocytosis: intracellular MSSA cells were less acidified, showing only a 1.1-fold decrease in pH, whereas MRSA cells experienced a 2-fold acidification. The data suggest that MSSA exhibit increased tolerance to acidic stress during phagocytosis. This resilience might be attributed to the preactivation of the acid tolerance response (ATR), a cellular defense mechanism initiated by exposure to mildly acidic conditions in either extracellular or intracellular environments.^22,25^ The reduced cytoplasmic pH of extracellular MSSA might have induced ATR, enabling these cells to better tolerate the harsh acidic environment inside phagolysosomes. This tolerance is likely based on pH-dependent gene regulation; mildly acidic pH modulates a large set of staphylococcal genes, including virulence, resulting in a cellular remodeling that adapts bacterial phenotypes to pH-variable environments.^102^

Our observations align with previous reports demonstrating that ATR enhances bacterial survival under lethal acidic conditions, including the strong acidic environment within macrophages.^25,48,103^ Previous research has shown that stress-resilient phenotypes that evolve in persister cells have a lower cytoplasmic pH.^31^ Similarly, our data indicate that more acidified MSSA are less affected by acid stress during phagocytosis, suggesting that low intracellular pH may serve as a predictive marker to help identify tolerant cells before stress exposure.

It is important to note that for this proof-of-concept study, we report population-averaged *I*_red_/*I*_blue_ ratios. However, CouCyCF_3_ **1b** allows for visualization of single-cell traits, providing the potential to analyze heterogeneity in stress adaptation at the individual cell level. Overall, these results demonstrate that CouCyCF_3_ **1b** can be used to track phagocytosis and visualize differences in the physiological state of clinical *S. aureus* strains in complex biological samples.

## Conclusion

We report a ratiometric fluorescent sensor specifically designed to study the intracellular pH of bacteria. The CouCy scaffold reacts fast and reversibly with OH^–^ ions and induces a ratiometric emission change that can be quantified as *I*_red_/*I*_blue_. The sensitivity of the scaffold was successfully adjusted by introducing EWG to the indoleninium core, resulting in CouCyCF_3_ **1b** having a biologically relevant, large dynamic sensing range (pH 5.0–7.5). We demonstrated live-cell pH sensing in *E. coli*, *S. epidermidis*, and clinical isolates of *S. aureus* using fluorescence microscopy and flow cytometry. Our probe has a higher sensitivity than the commercially available small-molecule BCECF-AM or the protein sensor pHluorin. This feature enabled the visualization of pH-sensitive *E. coli* cells, highlighting the ability to visualize single-cell phenotypes in real-time. Additionally, the phagocytosis of clinical *S. aureus* strains in THP-1 monocytes or blood samples could be monitored.

Our studies show that CouCyCF_3_ **1b** is a general pH sensing probe. Despite the high uptake and accumulation in bacteria, further improvements could be made regarding its retention in bacteria. To reduce efflux over time, functional groups for intracellular trapping could be installed, such as an isothiocyanate or an *N*-hydroxysuccinimide (NHS) ester, as used in many commercially available probes. CouCy probes are an excellent scaffold for bacterial imaging and a promising tool for detecting phenotypic heterogeneity, which, with further fine-tuning, might allow unraveling the physiological state of resistant or persistent bacteria of clinical relevance.

## Supplementary Material



## Notes

The contents of this file include experimental details, supplementary figures, and ^1^H and ^13^C NMR spectra (PDF)

## Notes

All the raw data from this paper are available on Zenodo at DOI: 10.5281/zenodo.17034283.
